# Pain rewarded: hyperalgesic and allodynic effect of operant conditioning in healthy humans—protocol for a systematic review and meta-analysis

**DOI:** 10.1186/s13643-018-0765-y

**Published:** 2018-07-17

**Authors:** Wacław M. Adamczyk, Kerstin Luedtke, Ewa Buglewicz, Przemysław Bąbel

**Affiliations:** 1 Pain Research Group, Institute of Psychology, Jagiellonian University, Kraków, Poland; 2 Department of Kinesiotherapy and Special Methods in Physiotherapy, The Jerzy Kukuczka Academy of Physical Education, ul., Mikolowska 72B, 40-065 Katowice, Poland; 3 Department of Orthopedics, University of Luebeck, Luebeck, Germany

**Keywords:** Allodynia, Hyperalgesia, Instrumental learning, Chronic pain, Behaviour, Somatoform disorders

## Abstract

**Background:**

‘Pain rewarded’ is a hypothesis wherein acute pain sufferers are exposed to reinforcers and punishers from their environment that shape their behaviour, i.e. pain responses. Such a point of view has been taken for granted by many clinicians and researchers although existing evidence has not yet been systematically summarized. This planned systematic review and meta-analysis is aiming to summarize the research findings on pain modulation (hyperalgesic effect) and pain elicitation (allodynic effect) resulting from operant conditioning procedures in healthy humans.

**Methods:**

The systematic review will be performed by searching for articles indexed in PubMed database, Cochrane Register of Controlled Trials (CENTRAL), Web of Science™, ScienceDirect, EBSCO database, PsycINFO, MEDLINE, PsycARTICLES and CINAHL. Studies will be included if they investigate healthy humans, exposed to modulation or elicitation of a pain experience induced by operant conditioning. Studies will be screened for eligibility and risk of bias by two independent assessors. Narrative and meta-analytical syntheses are planned.

**Discussion:**

Data will be pooled and analyzed qualitatively and quantitatively (if possible) in order to advance the understanding of pain mechanisms, especially the development of chronic pain. This systematic review will guide the planning of future experiments and research by summarizing important technical details of conditioning procedures in healthy humans.

**Systematic review registration:**

PROSPERO CRD42017051763

## Background

Chronic non-specific musculoskeletal pain is unrelated to the extent of tissue damage and does not seem to have any biological advantage in humans. It is a leading cause for suffering and disability worldwide [1] and has a high socioeconomic impact [2]. For this reason, chronic pain syndromes are in the focus of many experimental studies, but mechanisms underlying their origin and long-term maintenance are poorly understood [3–5]. Current concepts suggest that chronic pain can be developed as a result of learning processes, including Pavlovian [6, 7] and operant conditioning [8]. In contrast to Pavlovian conditioning, operant conditioning controls behaviours according to their consequences rather than their antecedents and is driven by reinforcers and punishers delivered in varied schedules, for instance, partial or continuous [9]. Current concepts of pain suggest that pain is a private and subjective experience that can be measured using valid instruments [10, 11]. Typically, pain is measured on a quantitative scale indicating its intensity. Thus, in this proposed systematic review and meta-analysis, pain will be treated as private behaviour and operationalized using pain ratings on a standardized scale measuring pain intensity.

Hitherto, there have been several more [12, 13] or less [14] effective attempts to control pain via operant conditioning in healthy humans. These attempts aimed to imitate the clinical environment in which patients are exposed to pain-related learning. A typical clinical example could be a patient with acute pain related to an injury that persists after tissue healing because pain behaviour has been rewarded or—using behavioral terms—reinforced. Fordyce [8] suggested that financial benefits or attention from others given to someone in pain are robust reinforcers influencing the maintenance of pain [15, 16]. To the best of our knowledge, studies addressing this model within an experimental setting [12–15, 17–20] have not yet been adequately summarized, neither qualitatively nor quantitatively. If pain can be evoked or maintained by operant conditioning in the absence of nociception or after the reduction of nociception, novel treatment strategies incorporating mechanism of operant conditioning should be applied at an earlier stage of a painful condition. We anticipate to find two separate lines of research: one is focused on operantly conditioned hyperalgesia and the other on conditioned allodynia. In the former, pain is enhanced although the stimulus intensity does not increase; in the latter, pain is maintained although the stimulus intensity is reduced below the pain threshold.

‘Pain rewarded’ is a hypothesis wherein acute pain sufferers are exposed to reinforcers and punishers from their environment that shape their pain experience. Such a point of view has been taken for granted by many clinicians and researchers although existing evidence has not been systematically summarized [21]. This planned systematic review and meta-analysis is aiming to summarize the research findings on pain modulation (hyperalgesic effect) and pain elicitation (allodynic effect) as a result of operant conditioning in healthy humans.

## Research questions

The following are the two research questions:

1. Can operant conditioning induce a hyperalgesic effect in healthy humans?
2. Can operant conditioning induce an allodynic effect in healthy humans?

## Methods

This review protocol was designed a priori according to the Preferred Reporting Items for Systematic Review and Meta-Analyses Protocols (PRISMA-P) guideline [22]. The systematic review protocol follows the recommendations on data searching and data processing described in the *Cochrane Handbook for Systematic Reviews* [23]. The record of this protocol was registered in the PROSPERO database (no: CRD42017051763).

### Search strategy for the identification of studies

We will use search terms referring to the population studied (P), operant conditioning as an intervention (I), and any measure of pain used in an experimental study as an outcome (O). The comparator (C) and study type (S) will be omitted in order to avoid exclusion of relevant studies. Instead, detailed selection criteria will be used to classify studies according to the comparator used and study type. Only studies published in English will be considered and evaluated in the review process. There will be no restrictions regarding the time frame of published articles. Both medical subject heading (MeSH) terms and natural language expressions will be combined for the search in electronic databases.

#### Search terms and phrases (PubMed database)

The following are the search terms and phrases:

1. healthy humans
2. healthy controls
3. pain-free controls
4. healthy patients
5. healthy participants
6. healthy persons
7. healthy subjects
8. healthy people
9. “Healthy Volunteers”[Mesh]
10. humans
11. #1 OR #2 OR #3 OR #4 OR #5 OR #6 OR #7 OR #8 OR #9 OR #10
12. punishment
13. “Punishment”[Mesh]
14. punisher*
15. reinforcer*
16. reinforcement learning
17. operant learning
18. instrumental learning
19. “Reinforcement (Psychology)”[Mesh]
20. reinforcement
21. operant conditioning
22. “Conditioning, Operant”[Mesh]
23. reward learning
24. operant
25. #12 OR #13 OR #14 OR #15 OR #16 OR #17 OR #18 OR #19 OR #20 OR #21 OR #22 OR #23 OR #24
26. pain
27. “Pain”[Mesh]
28. pain response
29. pain behaviour
30. pain behavior
31. pain perception
32. pain intensity
33. #26 OR #27 OR #28 OR #29 OR #30 OR #31 OR #32
34. #11 AND #25 AND #33

#### Databases

Nine databases will be searched for relevant articles including:

1. PubMed database
2. Cochrane Register of Controlled Trials (CENTRAL)
3. Web of Science™
4. ScienceDirect
5. EBSCO database
6. PsycINFO
7. MEDLINE
8. CINAHL
9. PsycARTICLES

#### Searching other resources

To extend the scope of the review, three additional sources of data will be searched: reference lists of identified articles will be screened for other relevant articles, contents of relevant pain journals will be hand searched and key authors in the field of pain and learning will be contacted for ongoing research or unpublished data.

### Selection of relevant studies

A list of inclusion and exclusion criteria is presented in Table 1. Studies will be included if they use an operant conditioning paradigm in which participants are exposed to noxious stimuli and in which their behaviour, i.e. pain, is rewarded and/or punished while the noxious stimuli are maintained/modulated over time. We anticipate two separate lines of research, one on conditioned hyperalgesic and the other on conditioned allodynic effects. In the former, pain is enhanced while stimulus intensity is constant; in the latter, pain is still perceived although the stimulus intensity is reduced below initial pain threshold. Such a distinction in studies on conditioned allodynia and hyperalgesia is in line with Madden et al. [6] in a meta-analysis on pain and classical conditioning. The second reason for this distinction is conceptual. In order to understand chronic non-specific pain as an experience that outlasts the normal nociceptive period, studies on conditioned allodynia are crucial.

**Table 1:** The list of inclusion and exclusion criteria

Issue	Inclusion criteria	Exclusion criteria
P	- Healthy controls - Pain-free controls	- Information on any disease
I	- Operant conditioning procedure	- Classical conditioning studies - Observational learning studies - Other interventions combined with operant conditioning
C	- No restriction - Within-subject control - Between-subject control - No intervention - Non-contingent learning - No conditioning procedure	N/A
O	- Pain measured on any pain scale	N/A
S	- Experimental studies - Randomized controlled trials - Published until 2018	- Conference proceedings - Only abstracts available

*P* participants, *I* intervention, *C* comparison, *O* outcome, *S* study type

Combining studies in this field will advance our understanding of pain syndromes without nociception and will promote the implementation of a learning perspective into the experience and modulation of pain. No restrictions related to the operant conditioning procedures apply. Studies can be included based on any type of reinforcers/punishers (e.g. verbal, visual) and reinforcement schedules (e.g. partial, continuous). Studies combining operant conditioning with other types of learning (e.g. classical conditioning) or procedures aiming to modulate pain (e.g. verbal suggestion of nocebo hyperalgesia) will not be included. Studies will be included if pain is measured on a pain scale (e.g. Numerical Rating Scale [NRS] or Visual Analogue Scale [VAS]).

Only studies recruiting healthy, pain-free participants will be included in the systematic review. Participants should be healthy and pain-free at the time of the experiment. Participants’ state will be treated as ‘healthy’ if it will be explicitly written in the article or if there will be no indication of disease. Studies will be included without restriction regarding the type of the control intervention. We plan to include studies in which within- or between-subject control interventions were used. This includes ‘no-intervention’, ‘non-contingent conditioning’ or an intervention without conditioning. In case of a sufficient level of homogeneity across included studies, data will be combined in meta-analyses.

### Data collection and analysis

#### Selection of studies

Two stages of study selection will be performed: preliminary and final. The preliminary study selection will be based on the title and abstract screening of the identified articles/publications by two independent raters according to the eligibility criteria. Subsequently, in the final stage, full-text articles of the remaining studies will be reviewed, using the same inclusion/exclusion criteria (Table 1). The agreement between the two assessors will be expressed using the Cohen’s kappa statistic, a statistical coefficient which is commonly used on nominal/categorical data [24]. In case of disagreement between the two assessors that cannot be resolved in a discussion, a third independent expert will be approached. The third person will decide whether the publication will be included in the further review process. This procedure follows the recommendations of chapter 7 of the Cochrane Handbook [23]. The selection process will be documented in a flowchart, as recommended by the PRISMA statement [25].

#### Risk of bias assessment

All included studies will be critically appraised regarding their potential risk of bias by two independent assessors. Randomized controlled trials and other experimental studies will be assessed by using the tool provided by the Cochrane Collaboration [23], as recommended by the PRISMA statement [26] and used in a previous meta-analysis on experimental pain-related studies [27]. The Cochrane risk of bias tool will be customized. As in a previous meta-analysis, ‘blinding of personnel’ and ‘blinding of outcome assessors’ will not be evaluated because it is not possible to blind researchers for self-reported outcomes. However, blinding of participants will be assessed based on the quality of the cover story used preceding the conditioning procedure. The item ‘other bias’ will be split into two categories and assessed separately, i.e. power analysis and contingency awareness. The latter category is required to reflect specific bias occurring in studies on operant conditioning. Not controlling for contingency awareness can induce bias caused by the participants’ awareness of undergoing a conditioning procedure.

The assessors’ agreement will be calculated using the Cohen’s kappa statistic. In case of disagreement that cannot be resolved in a discussion, the third independent assessor will arbitrate. The risk of bias judgement will be presented in a separate table.

#### Data extraction

Data will be extracted using a predesigned data extraction sheet. Extraction of descriptive data and outcome measures will be similar to those reported in a previous systematic review on Pavlovian conditioning [6]. Two independent researchers will extract the following items: sample size, age, gender, nature of noxious stimuli (e.g. electrical or thermal), equipment, body area exposed to pain, number of conditioning, baseline and extinction trials, intervals between trials, schedule of reinforcement, type of control intervention, form and type of reinforcers and/or punishers, contingencies, mean and standard deviation (SD) of main outcome measures (e.g. difference in pain intensity between experimental and control condition), type of pain scale, calibration procedure and intensities of stimulation used in the experiment. In case of disparity in the extracted data, final tables presenting study characteristics will be created as a result of discussion with co-authors of the review. Study authors will be contacted electronically if relevant data is missing from the identified studies, and all authors of the studies included in the meta-analysis will be contacted and asked to provide raw data. If the authors do not respond, two follow-up e-mails will be sent 1 week apart. If no response is noted after the second follow-up, no further contact will be attempted.

#### Data synthesis

All identified studies will be included in the qualitative synthesis and presented in predesigned tables. Whether a combination of the studies in a meta-analysis will be attempted, it will depend on the compatibility of the outcome measures and the level of bias across included studies. To increase the internal validity, studies with a high risk of bias in the category ‘blinding of participants’ will be excluded from the meta-analysis. If appropriate, random effect models will be calculated, and data will be pooled into meta-analysis. The effect size will be imputed into the analysis as Hedges’ *g*, i.e. standardized and unbiased mean difference between experimental and control condition and weighted by the sample size in the given study. Hedges’ *g* around 0.2 will be considered a small effect; a value around 0.5, medium effect; and 0.8 or larger as a large effect [27, 28]. All calculations will be performed using the Review Manager (RevMan) software from the Cochrane Collaboration (version 5.3. Copenhagen: The Nordic Cochrane Centre, The Cochrane Collaboration, 2014). Random effect models will be chosen due to the potential variability in the study design and conditioning procedures among included studies [29]. If possible, publication bias will be assessed by visual inspection of a funnel plot.

#### Further steps

The review will be prepared for publication in a scientific journal accepting articles in the field of pain science such as PAIN or The Journal of Pain. If any modification to this protocol is introduced, it will be clearly explained throughout the article.

## Discussion

This systematic review will summarize the literature on pain modulation and pain elicitation by means of operant conditioning. Data will be pooled and analyzed qualitatively and quantitatively in order to advance our understanding of pain mechanisms, especially of chronic pain. The results of the planned systematic review will guide future research by summarizing important technical details of conditioning procedures, such as the schedule of reinforcements, type of reinforcements/punishers, etc.
